# Astrocytic K^+^ regulation during neurodegenerative diseases

**DOI:** 10.3389/fnagi.2026.1782460

**Published:** 2026-03-19

**Authors:** Evgeniia Samokhina, Yossi Buskila

**Affiliations:** 1 Clem Jones Centre for Ageing Dementia Research, Queensland Brain Institute, The University of Queensland, Brisbane, QLD, Australia; 2 Laboratory of Cellular neurophysiology, School of Medicine, Western Sydney University, Campbelltown, NSW, Australia; 3 The MARCS Institute, Western Sydney University, Campbelltown, NSW, Australia

**Keywords:** ALS, Alzheimer's disease, astrocytes, homeostasis, neurodegeneration, potassium

## Abstract

Neurodegenerative diseases are a group of chronic, progressive disorders characterized by the gradual loss of neurons in specific areas of the central nervous system. Historically, a “neurocentric” paradigm viewed glial cells, such as astrocytes, as cells that provided adequate support for neuronal energy metabolism and controlled local cerebral blood flow. However, studies from the past two decades found that astrocytes are involved in synaptic function through different mechanisms, including the uptake of extracellular glutamate molecules and potassium ions following synaptic neuronal transmission. Also, astrocytes respond to neurotransmitters and neuromodulators through alterations of intracellular ion concentrations (e.g., Na^+^, Ca^2+^, K^+^) and the release of gliotransmitters. Astrocytes play a pivotal role in preserving potassium homeostasis within the central nervous system through their potassium channels, a process known as “potassium clearance.” Impaired astrocytic potassium clearance mechanisms can result in neuronal hyperexcitability, leading to increased glutamate release, overactivation of glutamate receptors, and cytotoxicity. Recent studies suggest that these factors can cause cell death and neurodegeneration, and further indicate a region-specific glial dysfunction in neurodegeneration, which reflects the heterogeneity of glial cell function and sensitivity across different brain regions. Overall, this manuscript offers novel insights into a relatively new concept that glial cells can actively shape neuronal activity and survival.

## Introduction

### Overview of neurodegeneration

Neurodegenerative diseases encompass progressive, debilitating conditions resulting from the progressive loss of neuronal structure or function within the central or peripheral nervous system. The loss of neurons in neurodegeneration and the collapse of the neural network lead to impairments in memory, cognition, behavior, sensory, and motor functions. According to the recent epidemiological analyses, the global burden of neurodegenerative disorders is rapidly increasing, with a pronounced rise expected by 2040, particularly in high-income regions (Hao and Chen, 2025; Liu et al., 2025). Regrettably, there is currently no effective disease-modifying treatment for neurodegeneration, and only symptomatic therapy is available.

Several pathological hallmarks of neurodegenerative diseases include synaptic dysfunction, aberrant proteostasis, altered energy metabolism, DNA/RNA abnormalities, and inflammation (Noori et al., 2021). Additionally, neurodegenerative diseases can be associated with protein misfolding or proteinopathy, resulting in protein toxicity or loss of physiological functions. This can lead to the generation of small oligomeric or large fibrillary aggregates, which lead to accumulation of intracellular or extracellular proteins that cause neurotoxicity and ultimately lead to neurodegeneration (Numaguchi et al., 2024; French et al., 2019; Robinson et al., 2018; Nascimento et al., 2018).

Amyotrophic lateral sclerosis (ALS) and Alzheimer's disease (AD) are neurodegenerative diseases that are distinguished by their distinctive clinical and anatomical characteristics. ALS is characterized by the progressive degeneration of both upper and lower motor neurons, resulting in muscle weakness, loss of motor control, and eventual paralysis and death. In contrast, AD is characterized by the loss of neurons in the hippocampus and association cortices, leading to memory impairment and cognitive decline. Although ALS and AD target distinct types of neurons and manifest distinct clinical symptoms, they share common molecular pathologies that may be the driving force behind the neurodegenerative processes in both diseases.

**Figure 1:**
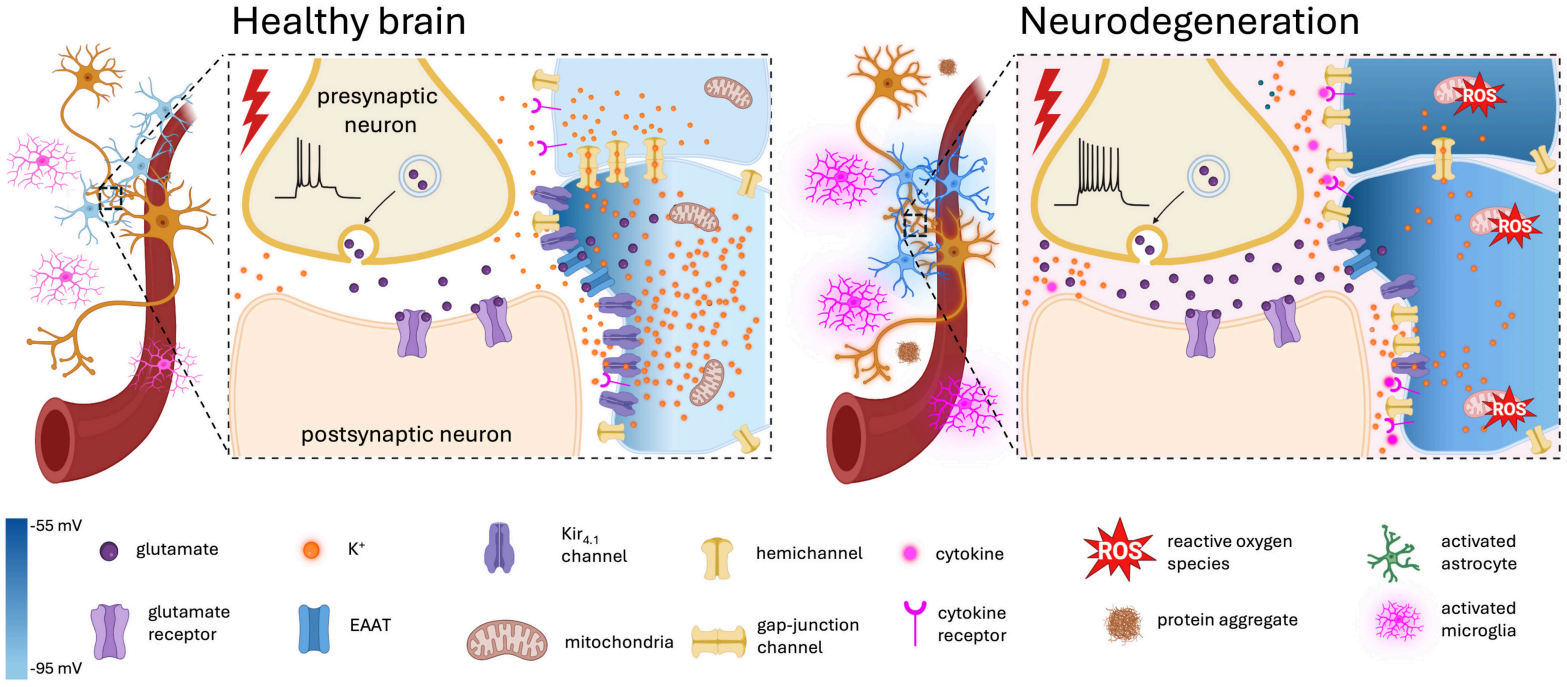
Astrocytic pathogenesis in neurodegeneration. Schematic illustration depicting the involvement of mitochondrial dysfunction and neuroinflammation in the dysregulation of potassium homeostasis during neurodegeneration. In AD and ALS, astrocytes exhibit impaired mitochondrial electron transport chain activity, leading to increased ROS production and ATP depletion. Activated microglia release cytokines and chemokines that promote a reactive astrocyte phenotype and downregulation of Kir_4.1_ expression. Reactive astrocytes also exhibit increased hemichannel expression and impaired gap junction coupling, resulting in impaired astrocytic syncytium and a diminished electrodiffusive driving force for K^+^ uptake. This leads to astrocytic depolarisation and reduced K^+^ clearance, causing extracellular K^+^ accumulation. Additionally, morphological changes in reactive astrocytes affect glutamate reuptake via EAAT's. Overall, the shift in the K^+^ gradient across neuronal membranes, as well as a decrease in glutamate uptake by EAAT's drives neuronal hyperexcitability and ultimately degeneration.

A common pathological hallmark of both ALS and AD is altered energy metabolism (Figure 1), mainly as there are sever mitochondrial impairments that lead to a decrease in oxidative phosphorylation and ATP production (Schweingruber et al., 2025; Beck et al., 2016; Do-Ha et al., 2018). In ALS, there is substantial evidence of mitochondrial morphological abnormalities, reduced activity of electron transport chain complexes, increased production of reactive oxygen species (ROS), and altered dynamics of mitochondrial fission and fusion, contributing to reduced ATP synthesis and neuronal dysfunction (Hor et al., 2021; Sasaki and Iwata, 2007). Moreover, a genetic study using CRISPR/Cas9 in iPSCs derived from an ALS patient carrying the L144FVX SOD1 mutation demonstrated that correction of the mutation by inserting wild-type SOD1 sequences restored SOD1-associated intracellular ATP levels and improved motor neuron survival (Imamura et al., 2017), highlighting energy failure as a shared pathological driver across ALS forms. Similarly, AD brains exhibit mitochondrial bioenergetic deficits characterized by disrupted oxidative phosphorylation, decreased mitochondrial enzyme activity, mitochondrial DNA damage, and elevated oxidative stress driven by amyloid-β and tau pathology (Beck et al., 2016; Calvo-Rodriguez et al., 2024). These mitochondrial dysfunctions correlate with impaired neuronal glucose metabolism and account for the brain energy hypometabolism detected decades before cognitive symptom onset (Mosconi et al., 2010; Popuri et al., 2021).

### Neuroinflammation during neurodegeneration

Neuroinflammation is another prevalent pathological mechanism of neurodegeneration that is common in both AD and ALS. It is defined as an inflammatory response within the brain or spinal cord, and is mediated by the production of cytokines, chemokines, reactive oxygen species, and secondary messengers. The neuroinflammatory factors are produced by resident CNS glia (microglia and astrocytes), endothelial cells, and peripherally derived immune cells (Kekesi et al., 2019). While the inflammatory response can have beneficial effects by promoting tissue repair and removing cellular debris, sustained chronic inflammatory responses are detrimental, can inhibit regeneration and promote neurodegeneration (Gamage et al., 2023). Indeed, previous studies conducted on animal models for ALS have demonstrated that activated microglia and astrocytes secrete high levels of proinflammatory factors, including TNF-α, IL-1β, and IL-6, thereby directly contributing to motor neuron toxicity and synaptic dysfunction (Xiao et al., 2007; Zhao et al., 2010). Consistent with these findings, recent research has identified a correlation between decreased regulatory T lymphocytes and disease progression and severity in ALS patients (Beers et al., 2017). Furthermore, ALS patients exhibit a distinct proinflammatory signature highlighting increased cytoplasmic NF-κB in glia due to TDP-43 burden, underscoring altered neuroinflammatory signaling in ALS (Rifai et al., 2023). Similarly, AD pathogenesis is closely linked to the activation of microglia and astrocytes. This activation generate a positive feedback loop of cytokine release, including IL-1β, IL-6, and TNF-α that exacerbates amyloid pathology and promotes neuronal loss (Zheng et al., 2016; Swardfager et al., 2010). Moreover, chronic activation of p38 MAPK and NF-κB signaling pathways amplifies cytokine production, tau phosphorylation and synaptic impairment, further suggesting a key role of inflammation in AD pathogenesis (Munoz et al., 2007).

Genetic studies also support a neuroinflammatory component of neurodegeneration in AD. Notably, variants in immune-modulating genes, such as TREM2, affect microglial responsiveness and substantially elevate the risk of AD (Jonsson et al., 2013; Guerreiro and Hardy, 2013). Taken together, these findings illustrate a unifying pathological cascade across AD and ALS. Protein aggregation, dysregulated glial activity, compromised immune homeostasis, and persistent cytokine signaling form a shared framework that accelerates neuronal degeneration in both neurodegenerative diseases.

### Astrocytic dysfunction during AD and ALS

Astrocytes in AD and ALS undergo profound morphological remodeling that reflects their transition from a homeostatic to a reactive state (Rossi and Volterra, 2009; Stevenson et al., 2020; Filipi et al., 2024; Kim et al., 2024). This transition is not uniform and driven by pathological cues. In AD, astrocytic remodeling includes hypertrophy, particularly in regions surrounding amyloid-β (Aβ) plaques. This morphological response is spatially heterogeneous: astrocytes adjacent to plaques show pronounced hypertrophy, marked by the thickening of primary processes and increased expression of GFAP and vimentin (Habib et al., 2020), whereas those in more distal regions often display atrophy and reduced territorial domains, suggesting diminished neuronal support (Endo et al., 2022; Rodriguez et al., 2023). Central to these structural changes is dysregulated intracellular calcium dynamics that impair astrocyte-to-neuron communication and contribute to early network hyperexcitability in AD (Shah et al., 2022). Aβ exposure directly induce spontaneous aberrant Ca^2+^ transients in astrocytes. This further drives calcium-dependent cytoskeletal remodeling through the activation of calcineurin (Sompol et al., 2017), resulting in transient morphological plasticity that may represent an early compensatory adaptation to contain plaque spread, which ultimately fails as Aβ accumulates (Rowland et al., 2025). Morphological changes in astrocytes, including process retraction and territory shrinkage, also correlate with impaired glutamate clearance (due to a decreased expression and function of glutamate transporters such as EAAT1) (Mishra et al., 2013), altered activity of the glymphatic system (which further impair the clearance of endogenous and exogenous Aβ-42) (Liang et al., 2020; Xia et al., 2017), metabolic coupling, and potassium buffering (Bellot-Saez et al., 2018, 2017).

Astrocytes in ALS follow a similar dynamic, yet disease-specific morphological trajectory. Early in the disease, astrocytes exhibit hypertrophic reactivity marked by enlarged cell bodies, thickened processes, and elevated expression of complement proteins and other inflammatory markers (Rossi et al., 2008). Similar to AD pathogenesis, these structural alterations correlate with downregulation of glutamate transporters, impaired metabolic support to motor neurons and a decrease in K^+^ clearance rate (Foran and Trotti, 2009; Stevenson et al., 2023). Single-cell and spatial transcriptomic analyses in both AD and ALS reveal diverse astrocyte subpopulations with distinct morphological and molecular profiles that vary with disease stage, brain region, and local microenvironmental signals (Habib et al., 2020; Serrano-Pozo et al., 2024; Liu et al., 2020; Clarke et al., 2021; Taha et al., 2022). For example, in AD, a specific disease associated subpopulation of astrocytes that are characterized by the upregulation of genes involved in lipid metabolism and proteostasis, such as CTSB and ApoE appears at the onset of cognitive decline and increase in abundance with age (Habib et al., 2020). Similarly, in ALS, regional differences in astrocyte reactivity between the motor and sensory cortices (Stevenson et al., 2023), as well as spinal cord and motor cortex (Kelley et al., 2018; Maniatis et al., 2019), suggest that local microenviroment dictate the extent of morphological alterations. Specifically, the presence of mSOD1 (Kelley et al., 2018) or TDP-43 aggregates (Stringer and Weiss, 2023) triggers a loss of essential homeostatic proteins (like Kir_4.1_) that varies significantly between regions (Olsen et al., 2007). Together, these findings position astrocytic morphological remodeling as both a marker and mediator of neurodegenerative progression. The dynamic interplay between hypertrophy, process retraction, and eventual atrophy impairs essential astrocytic functions and creates conditions that increase the susceptibility of neurons to damage and degeneration.

### Alterations in astrocytic K^+^ clearance during neurodegeneration

Astrocytes play a key role in the regulation of K^+^ homeostasis via dynamic modulation of potassium clearance from the extracellular milieu (Bellot-Saez et al., 2017, 2021; Buskila et al., 2019; Kaiser et al., 2006). Recent studies have elucidated pronounced deficits in astrocytic potassium clearance during the progression of ALS and AD (Liu et al., 2025; Stevenson et al., 2023; Samokhina et al., 2025; Huffels et al., 2022). Notably, studies conducted on animal models for ALS identified a progressive decline in the expression of Kir_4.1_ channels in the spinal cord (Kaiser et al., 2006) and markedly reduced potassium influx in cortical astrocytes, indicating a widespread loss of potassium regulation capacity in ALS-affected tissues (Kaiser et al., 2006; Bataveljić et al., 2012). These findings were corroborated by direct measurement of the K^+^ clearance rate in SOD1 mouse model for ALS, which indeed indicate a region-specific decrease in the motor cortex, primarily attributed to a decreased influx of K^+^ through Kir_4.1_ channels and reduced astrocytic connectivity (Stevenson et al., 2023), and volume alterations (Filipi et al., 2024). While potassium dysregulation in ALS is predominantly confined to motor pathways, it manifests altered distribution in AD. A recent study indicate a substantial reduction in astrocytic K^+^ clearance in the hippocampus of 5xFAD mouse model for AD (Samokhina et al., 2025). This decrease was associated with Kir_4.1_ channel dysfunction and a diminished astrocytic network. Consistent with these findings, (Ding et al., 2024) observed elevated extracellular K^+^ levels in the CSF of mouse model for AD, accompanied by downregulation of potassium channels. Notably, a reduction in the expression of Kir_4.1_ channels was also observed in the postmortem brains of Alzheimer's disease patients (Wilcock et al., 2009). However, a recent study on APP/PS1 mouse model indicated that the expression of Kir_4.1_ channels seems to increase in the vicinity of amyloid-β plaques, suggesting that Kir_4.1_ regulation in AD may reflect spatial and temporal heterogeneity (Huffels et al., 2022), or may represent a compensatory response to increased local neuronal hyperactivity and elevate K^+^ levels (Bataveljic et al., 2024). This pattern may reflect a dynamic progression from localized compensation around the plaques to a widespread failure of homeostatic mechanisms. The molecular mechanisms underlying Kir_4.1_ downregulation in neurodegenerative conditions are likely multifactorial and closely linked to inflammatory and metabolic stress signaling (Arisi et al., 2015). Pro-inflammatory cytokines such as TNF-α and IL-1β, which are elevated in both AD and ALS, activate intracellular cascades including NF-κB and MAPK pathways (Lawrence et al., 2023). Activation of these transcriptional regulators, as well as DNA methylation, have been shown to alter astrocytic gene expression programs and may suppress KCNJ10 transcription, the gene encoding Kir_4.1_ (Zurolo et al., 2012; Boni et al., 2020). Astrocytic phenotypic transitions toward reactive states may further reshape membrane channel composition, prioritizing inflammatory signaling over homeostatic buffering functions (Li et al., 2019). The reduced expression of Kir_4.1_ channels in astrocytes compromises their ability to clear potassium and glutamate, leading to an increase in neuronal excitability (Tyurikova et al., 2025). Moreover, a recent study proposed that the simultaneous downregulation of Kir_4.1_ and upregulation of aquaporin-4 (AQP_4_) destabilizes astrocytic endfeet, disrupting ionic and water homeostasis (Abbasian et al., 2025). These studies collectively demonstrate that Kir_4.1_ deficiency in glial cells leads to widespread ionic imbalances, promoting neuronal hyperexcitability and neurodegeneration.

Astrocytic gap-junction coupling also facilitate the potassium clearance process in glial cells (Bojovic et al., 2025), however recent data indicate that this network undergoes progressive alterations in both ALS and AD. A recent study indicated an upregulation of connexin-43 (Cx-43), the predominant gap-junction protein expressed in astrocytes, in postmortem analysis of the spinal cord and the motor cortex of ALS patients (Almad et al., 2016, 2022). However, while the overall expression of Cx-43 increases, there is a functional shift toward hemichannel formation (Retamal et al., 2007), which negatively affects the formation of gap junctions and, consequently, the overall connectivity within the astrocytic syncytium. It is important, however, to note that Cx43 assembles into two functionally distinct types of channels: gap-junction channels and hemichannels. Structurally, gap junctions are formed by the docking of two hemichannels from adjacent cells to create a continuous intercellular channel, whereas hemichannels are single, undocked units that directly connect the cytoplasm to the extracellular space. Unlike gap junction channels, hemichannels mediate ATP release from astrocytes (Madeira et al., 2021), which activate P2X and P2Y receptors on microglia and thus promoting the release of neuroinflammatory factors (Imraish et al., 2023). A similar increase in Cx-43 expression has been found in AD models and patients (Huffels et al., 2022; Su et al., 2025). Functional studies that distinguish gap junctions from hemichannels revealed a reduction in biocytin-labeled astrocytic syncytium size in both ALS and AD models, indicating that astrocytic connectivity is compromised despite overall Cx43 upregulation (Stevenson et al., 2023; Samokhina et al., 2025; Angeli et al., 2020; Axelsen et al., 2013; Garré et al., 2010; John et al., 1999).

Together, these findings highlight a shared pathogenic mechanism across AD and ALS: astrocytic failure to maintain K^+^ homeostasis through Kir4.1 disruption and gap-junction compromise. The resulting imbalance amplifies neuronal excitability, accelerates synaptic stress, and contributes directly to the progression of ALS, AD, and potentially other conditions marked by glial dysfunction.

## Conclusion

Conventionally, research on neurodegenerative diseases has largely adopted a neurocentric approach, focusing on neuronal toxicity and protein aggregation as primary drivers of pathology. However, accumulating evidence supports a complementary gliocentric perspective in which astrocytic dysfunction actively shapes neuronal excitability, metabolic stability, and survival. In this context, impaired astrocytic K^+^ homeostasis emerges as a mechanistically unifying contributor to both AD and ALS. Disruption of Kir_4.1_ mediated K^+^ buffering and altered astrocytic gap-junction coupling compromise the ability of astrocytes to maintain extracellular ionic balance, which promote network hyperexcitability (Bellot-Saez et al., 2017; Buskila et al., 2019). Chronic hyperexcitability increases synaptic stress, enhances glutamate release, and exacerbates excitotoxic vulnerability particularly in already metabolically compromised neuronal populations (Cellot and Ballerini, 2025; Tok et al., 2022). Importantly, these ionic disturbances do not occur in isolation but interact with mitochondrial dysfunction, oxidative stress, and neuroinflammatory signaling, forming a self-reinforcing pathogenic loop that accelerates neurodegeneration.

One of the biggest conundrums in the study of neurodegenerative diseases is the unique resilience of neurons in specific brain regions to neurodegeneration, which arises from an interplay of region-specific gene expression, glial support, enhanced proteostatic capacity, and synaptic preservation (Mrdjen et al., 2019). Emerging transcriptomic and functional studies indicate that astrocytic subpopulations differ in baseline Kir_4.1_ expression, gap-junction connectivity, metabolic support capacity, and inflammatory responsiveness (Endo et al., 2022; Batiuk et al., 2020; Bayraktar et al., 2020). Such intrinsic diversity may determine whether astrocytes mount effective compensatory responses or undergo maladaptive transitions under pathological stress. Thus, regional differences in astrocytic resilience could shape the spatial progression of degeneration in AD and ALS.

From a therapeutic perspective, astrocytic K^+^ regulation represents a promising yet underexplored target. Strategies aimed at stabilizing Kir_4.1_ expression or function, modulating connexin-43 hemichannel activity to preserve intercellular coupling, or restoring astrocytic membrane polarization may help reestablish ionic homeostasis and reduce network hyperexcitability. However, the temporal dimension of astrocytic responses must be carefully considered, as early compensatory changes may transition into chronic maladaptive states. Future studies integrating electrophysiology, single-cell transcriptomics, and disease-stage–specific interventions will be essential to define when and how astrocyte-targeted therapies could be most effective.

In summary, impaired astrocytic potassium buffering is not merely a secondary consequence of neurodegeneration but may actively amplify disease progression. Recognizing astrocytes as dynamic regulators of neuronal excitability and network stability provides a broader framework for understanding selective vulnerability and opens new avenues for therapeutic development in AD, ALS, and potentially other neurodegenerative conditions.
